# Interprofessional Collaborative Relationship-Building Model in Action in Primary Care: A Secondary Analysis

**DOI:** 10.3389/fresc.2022.890001

**Published:** 2022-05-31

**Authors:** Pamela Wener, Leanne Leclair, Moni Fricke, Cara Brown

**Affiliations:** ^1^Department of Occupational Therapy, College of Rehabilitation Sciences, Faculty of Health Sciences, University of Manitoba, Winnipeg, MB, Canada; ^2^Department of Physical Therapy, College of Rehabilitation Sciences, Faculty of Health Sciences, University of Manitoba, Winnipeg, MB, Canada

**Keywords:** primary care, qualitative research, secondary analysis (qualitative), interprofessional collaboration, team-based care delivery

## Abstract

**Introduction:**

Team-based care has been integrated into primary care (PC) across Canada because it improves patient safety, effectiveness, efficiency, person-centredness, and equity. However, this integration in and of itself may not lead to improved patient care without effective interpersonal relationships amongst team members. Currently, teams have few tools to guide the development of collaborative relationships. The Interprofessional Collaborative Relationship-building Model (ICRB) was developed to be a tool for understanding the stages of development of the interprofessional team's relationship-building.

**Purpose:**

This qualitative secondary data analysis illuminates a PC team's experiences of their developing interprofessional relationships with occupational therapists and physical therapists who joined the PC team.

**Method:**

Eleven team member interviews of one primary care team from a family medicine teaching clinic affiliated with a training university and the health region in central Canada were analyzed using secondary data analysis. The team included family physicians (*n* = 4), nurses (*n* = 2), a social worker (*n* = 1), a mental health counselor (*n* = 1), occupational therapists (*n* = 2), and a physical therapist (*n* = 1). We used the ICRB for directed content analysis using the phased approach that includes the three main steps of data preparation, data organization and data presentation.

**Results:**

This team experienced the ICRB stages of Looking For Help, Fitting-In, and Growing Reciprocity thereby learning about one another to better understand what OT and PT may bring to the PC setting. However, contrary to the ICRB, co-location, was the context within which the collaborative relationship-building took place rather than a distinct developmental stage. Although team members did experience some level of Growing Reciprocity, this developing team had not yet established collaborative leadership processes. As the ICRB originally posited, communication and patient focus facilitated all stages of the relationship-building process and helped the team develop shared values and role clarity that establish how different team members contribute to improving quality care.

**Conclusions:**

The context of co-location with a patient focus and open communication facilitated the team's development with the occupational therapists and physical therapist. Collaborative leadership is a worthy goal for future research and clinical focus as it has implications for improving overall patient quality care and team member work satisfaction.

## Introduction

**Figure 1:**
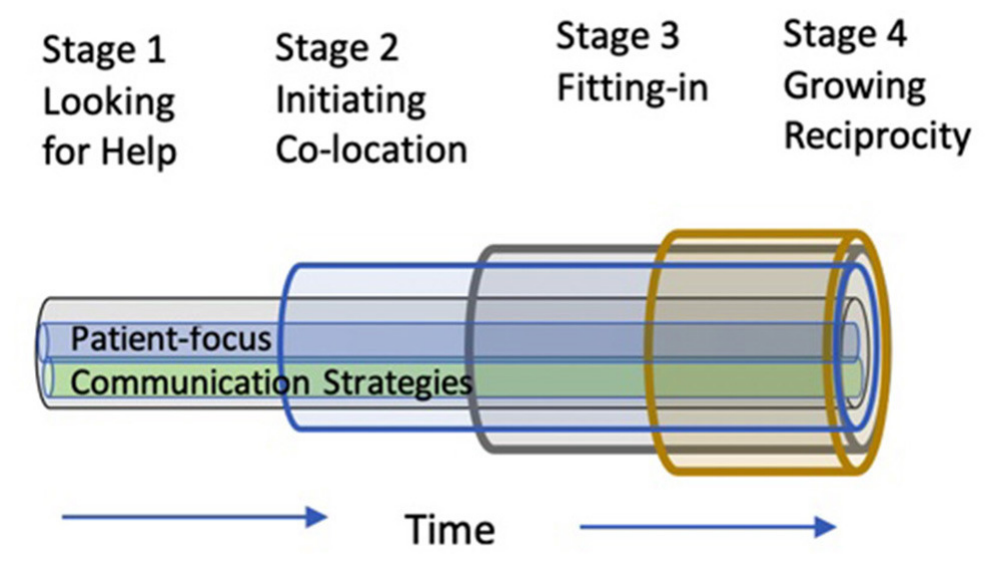
Interprofessional collaborative relationship-building model (ICRB).

Team-based care has been integrated into primary care across Canada because it improves patient safety, effectiveness, efficiency, person-centredness, and equity, where primary care is defined as the point of first contact with the public healthcare system (1). However, this integration in and of itself may not lead to improved patient care due to poor interpersonal relationships amongst team members and between providers and patients (2–4). Currently, primary care providers and patients have few tools to guide the collaborative relationship development process. Wagner's widely accepted primary care Chronic Disease Management model (5) emphasizes “interaction” or interpersonal relationships as foundational to collaborative relationship development (6). However, few collaborative practice models focus on interpersonal relationship elements such as willingness to collaborate, mutual trust, and respect amongst providers and between providers and patients (7). This study will explore the applicability of one such model in primary care—the Interprofessional Collaborative Relationship-Building Model (ICRB) (Figure 1) (8).

The ICRB model describes the processes used by primary care providers to develop their interprofessional relationships. The model includes four stages: (1) **Looking for Help:** where team members recognize a need for collaboration and the primary care providers recognize that another can fulfill the identified need; (2) **Initiating Co-Location**, where the primary care providers are in one physical location, increasing opportunities for face-to-face communication; (3) **Fitting-In**, where the collaborator shares skills and knowledge that fulfill the need identified in stage 1, thereby meeting the patient and team's needs; and, (4) **Growing Reciprocity**, where team members seek each other's perspectives, appreciating their similarities and differences for what they contribute to quality patient care. The ICRB also includes two central processes: (1) **Communication strategies**, how teams use formal and informal communication strategies, and (2) **Patient-focus**, whereby the patient is the consistent focus of the team's collaboration.

Over the last two decades, publicly funded primary care settings in Canada have begun to include other service providers such as psychologists and social workers (9, 10). The extent of integration of occupational therapists (OTs) and physical therapists (PTs) varies across Canada. For example, OTs and PTs in Ontario have been on primary care teams for over a decade, and it is estimated that over 70 OTs (C. Donnelly, personal communication, April 3, 2021) and over 90 PTs currently work in publicly funded primary care settings in Ontario (11). In contrast, Manitoba began to integrate OTs and PTs in 2013, with 15 OTs and five PTs currently in publicly funded primary care settings. Other provinces such as British Columbia, Alberta, and Nova Scotia are in the early stages of integrating OTs and PTs into primary care settings.

A national survey of OTs conducted in 2016 explored the integration of occupational therapy into primary care across Canada and the roles therapists were assuming in this practice context (12). Findings from this survey (*N* = 52) found that a growing number of OTs were being integrated into primary care, and that the majority (98%) were part of a team typically made of up of physicians, nurses, social workers, and dietitians. PTs in Canada have provided strong rationale for their inclusion in primary care to alleviate the burden of care from family physicians and provide access to many Canadians who currently cannot access a publicly-funded physical therapy (13).

While there is increasing evidence that OTs and PTs are joining primary care interprofessional teams, little is known about how these teams develop their interpersonal relationships with OTs and PTs in order to ensure high quality care. The purpose of this qualitative secondary data analysis was to explore a primary care team's perceptions of their experiences of interprofessional relationship-building with OTs and PTs who joined the primary care team. More specifically, we wanted to understand:

- How have the OTs and PTs developed interprofessional relationships as new members of the team?
- How have the other team members developed interprofessional relationships with the OTs and PTs?
- How could the primary care team further facilitate their team-building *via* their relationship-building?

## Methods

### Study Design

To answer the research questions, we conducted a qualitative secondary data analysis where the researcher reanalyzes data that was previously collected (14). Quantitative secondary data analysis of statistical data has been a common study design for over 50 years (14) whereas qualitative secondary data analysis, is a more recent research approach (14–17). Researchers use secondary data analysis to answer research questions that are related but distinct from the original purpose for the data collection (15, 17–19). The researchers of our original study collected interview data to understand the value of integrating OTs and a PT into a primary care clinic (20). The same group of researchers used the same data to explore the application of the ICRB and to understand the team's development. One of the advantages of secondary data analysis is that the researchers may gain a different understanding, often a nuanced aspect of a process (18) such as team development. Thorne referred to this type of secondary analysis as “analytic expansion” where the researcher aims to expand on the original research question [(21), p. 397]. Secondary data analysis design provides the advantage of delving deeper into the data without needing to intrude on participants to interview them again (21) while having the researchers from the original study involved in analysis may guard against misrepresentation of the interviews (17). Qualitative research approaches often aim to build theories and models, secondary data analysis “provides a means by which to move beyond that which is limited to a distinct sample and context to that which may begin to represent a more general claim.” [(21), p. 398] A final advantage of qualitative secondary analysis is that it tends to be cost-efficient because the data collection is previously done.

### Setting

The interviewed team worked in a family medicine teaching clinic affiliated with the training university and the health region in central Canada. At the time of the study, the clinic team included one full-time family physician, eight part-time family physicians, two primary care nurses, four physician assistants, a full-time social worker, and part-time pharmacist, dietitian, mental health counselor, child psychologist and consulting psychiatrist. Starting in January 2013, two days per week of occupational therapy (provided by two different OTs) and physical therapy services were added to the clinic. Fifteen first year family medicine residents spent 20 weeks per year training in the clinic. The residents worked under the supervision of the family physicians, and there was an attempt to expose residents to all the professions in the primary care team. The family physicians and/or residents were generally the first contact for the patients, and generated referrals to other professionals on the team, although interprofessional team members could also cross-refer. At the time of the study, all non-physician professionals were co-located in the clinic, and invited to participate in weekly administrative meetings (attendance varied due to part-time positions of non-physician staff) and a weekly case conference meeting that was primarily for resident teaching purposes. Frequency and length of interactions between the OTs and PT and the team's physicians were variable based on informal consultation and referral initiation of physicians.

### Data Collection and Analysis

The researchers of the original study invited all primary care team members from one clinic to participate in a 60-min individual interview (20). The original data was collected by a research assistant who received training in qualitative interviewing and the first author, an experienced qualitative interviewer. (PW) Interviews were audio recorded, transcribed verbatim by a trained transcriptionist and anonymized during transcription process.

Researchers used directed content analysis (22) to deductively analyze the data using an already developed theory or model. In classical qualitative research, coding is typically conducted inductively where the coding frame is created from the data (23). In a directed content study, a previously created model such as the ICRB (2016) is used as a coding frame and the data coded according to the model. Directed content analysis is appropriate for demonstrating and validating an existing theory or model (23). This process of directed content analysis may also provide new insights about the model (23). The researchers followed the three-phase (Preparation, Organizing, and Reporting), 16- step secondary analysis process outlined by Assarroudi [(24): Appendix A] and used the COREQ checklist (25) to ensure a high quality study and appropriate reporting (Appendix B).

#### Phase 1, Preparation

All authors of the manuscript had expertise in qualitative data analysis, selecting the sampling strategy, deciding on the focus and unit of the analysis, and immersing in the data. The first and last authors (PW and CB) used the verbatim interview transcripts of team members with the unit of analysis being the team. Resident focus groups from the original study were not included in this analysis as residents spent a limited time with the primary care team. PW and CB immersed themselves in the data exploring their utility for directed content analysis.

#### Phase 2, Organization

**Figure 2:**
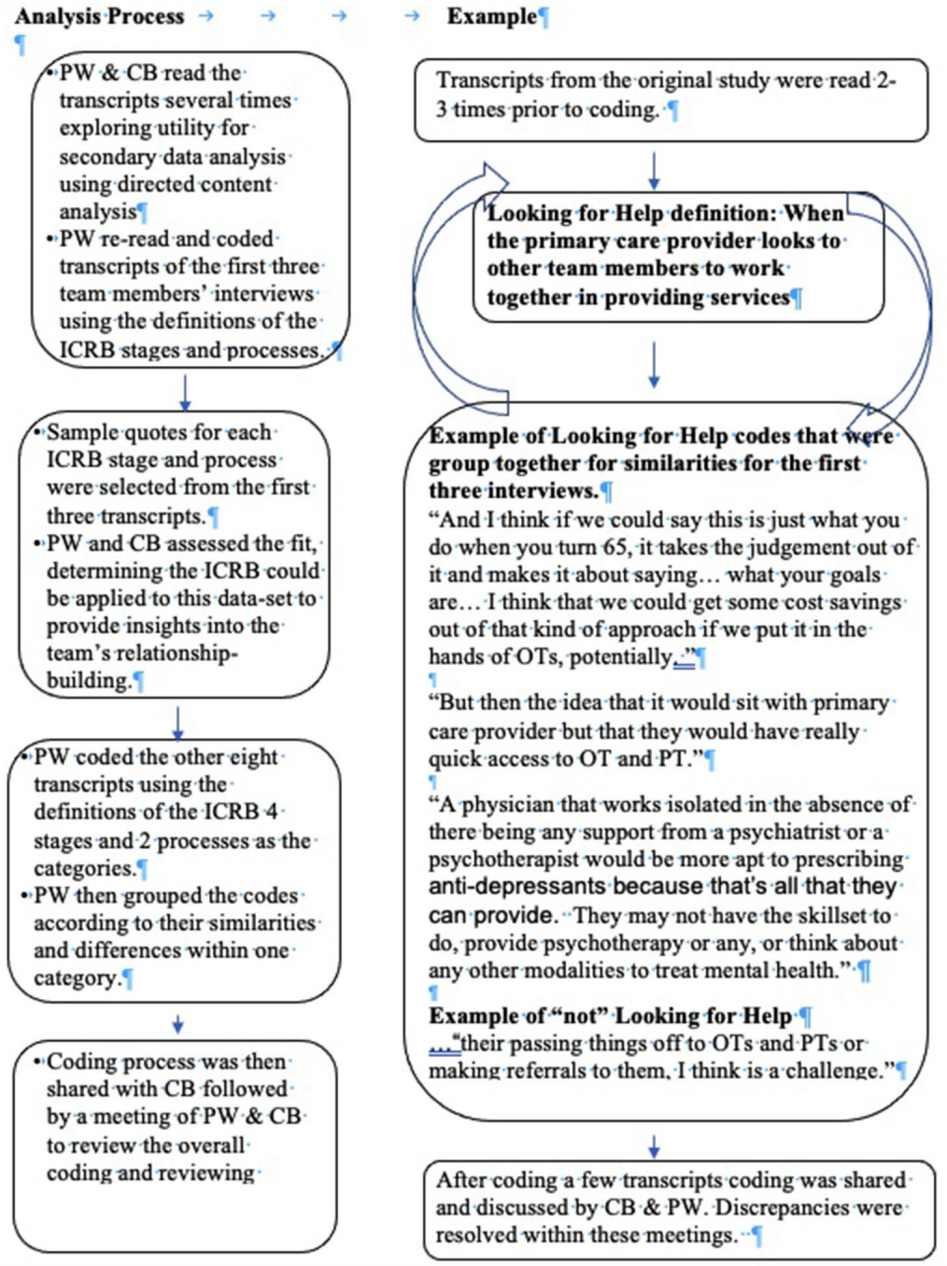
Example of secondary data analysis process: looking for help.

The first and last author (PW and CB) used the ICRB (8) four stages two processes, and theoretical definitions for the analytic matrix. Each transcript was read multiple times and coded, data were then transferred to the appropriate stage or process section. To pre-test the analytic matrix, PW coded transcripts of the first three team members' interviews. PW and CB assessed the fit, determining the ICRB could be applied to this data-set to provide insights into the team's relationship-building. Sample quotes for each ICRB stage and process were selected from the first three transcripts. Next, the main data analysis was performed, coding meaning units related to the study questions and then grouping these codes according to their similarities and differences to create generic categories. Last, links between generic categories and main categories were made. Figure 2 provides a visual of the analysis process and an example of some of the codes from one category, Looking for Help. The University of Manitoba Health Research Ethics Board provided ethical approval for this study (HS17336).

## Results

The 11 interviews were held with physicians (*n* = 4), a social worker (*n* = 1), a mental health counselor (*n* = 1), nurses (*n* = 2), OTs (*n* = 2) and a PT (*n* = 1). To protect the confidentiality of the participants, the nurses, the social worker and the mental health counselor will be referred to as “team member” in this paper. OTs and PTs will be identified as OT/PT and physicians will be identified as physicians. Details about the study participants provided in Table 1. Study participants had worked at the study primary care site for a minimum of one year. One participant, a family physician had no previous experience collaborating with OTs or PTs. Excluding the OTs and PT who participated in this study, four of the eight team members had previous experience collaborating with therapists while, three out of eight team members previous experience collaborating with PTs primarily or only.

**Table 1:** Participant demographic information.

**Profession**	**Gender**	**Years worked at study setting**	**Previous work settings**	**Previous collaboration with OTs and PTs**
Family physician	M	3	Emergency department, rural	Yes
Family physician	M	4	Fly-in physician	Primarily PT
Family physician	M	4	Hospice, personal care home, rural, urban community clinics, physician manager in urban hospital, university health services, women's health clinic	Yes
Family physician	F	4	Teaching primary care clinics, urban community health clinics, university health services	No
Dietitian	M	3	Acute care hospital, inpatient and outpatient rehabilitation setting	Yes
Social worker	F	4	Non-profit social services organization	Yes
Nurse	M	3	Community health clinic, remote communities	PT
Nurse	F	1	Acute care hospital	PT
Physical therapist	F	2	Acute care hospital, outpatient clinics, community health setting	Yes
Occupational therapist	F	2	Inpatient acute care, multiple sclerosis clinic, personal care home, rheumatology outpatient	Yes
Occupational therapist	F	2	Community pediatrics, community mental health, inpatient acute care, rural home care	Yes

The results describe and provide examples of how this team developed their interprofessional relationships in three of the four stages of the ICRB: **looking for help, fitting-in** and **growing reciprocity**. However, **co-location**, was not a distinct developmental stage in these data as it is presented in the ICRB. Rather **co-location** was the context within which the collaborative relationship-building took place. The team in this context experienced the stages of **looking for help, fitting-in**, and **growing reciprocity** thereby learning about one another to better understand what occupational therapy and physical therapy may bring to the primary care setting. As the ICRB originally posited, **communication** and **patient focus** facilitated all stages of the relationship-building process. **Communication** and **patient focus** are not discussed separately, but woven into the presentation of the results of the other themes. Some quotes have been shortened using ellipses, however we have not altered the meaning of quotes.

**1. Co-location as context** described the advantages that the team experienced working in the same physical environment as the OTs and PT and how co-location contributed to developing interprofessional relationships. The team took advantage of being co-located to have face-to-face **patient-focused** conversations to learn about one another and offer services they were previously unable to offer. For example, one of the members of the team was seeing a patient whose finances and health decisions were managed by a public trustee. The patient and the team member wanted to work toward having this guardianship removed. In the context of co-location, the team member shared this concern with the OT/PT in an informal conversation. Using their physical proximity, the team member described how the physician, team member, OT and patient worked together to reassess the patient's competency: “she, (the OT) came up with an assessment. Her and I and the physician met with the patient and told him what we thought. And so, she was just amazingly instrumental in that.” (Team Member 1).

The team members noted that formal communication with the OTs and PT does not necessarily require co-location since much of the communication is in written form using the electronic medical record. For example, the physicians used the electronic medical record (EMR) for referrals to the OTs and PT, and the therapists used the EMR to send reports after they had seen patients. This type of communication was seen as valuable since the written reports from the therapists helped the physicians better understand the occupational therapy and physical therapy roles in primary care. However, co-location helped the primary care team understand that face-to-face communication was beneficial for more complex communication that is required in getting to understand another professional's perspective, and addressing the needs of patients with complex situations. This face-to-face communication became more important as the team members moved further along the stages of the ICRB. In this quote, this team member is sharing how face-to-face communication allowed for clarification and improved understanding of referral appropriateness of a specific patient, “…like it may be a soft possibility in your mind but once you talk to someone, it's like, “Oh yeah, no, or …I'd love to see a person for that….” (Team Member 3)

Another unique advantage of the co-location of the OTs and PT in addition to communication was the perceived opportunity for the family practice residents to observe the therapists doing **patient-focused** assessments, clinic and home visits. The physicians appreciated the opportunity for their learners to understand the patients from multiple perspectives, giving the residents a comprehensive understanding of patient needs, “They (residents) see the therapy side of their visit and assessment and sometimes back-to-back with a medical visit that happens at the same (time). So they, residents, see both viewpoints of the same problem.” (Physician 3)

The context of co-location was a facilitative component of **looking for help, fitting-in** and **growing reciprocity**.

**2. Looking for Help** for this team was a stage in the ICRB where the already established primary care team looked to the newer members of the team (OTs and PT), to fill gaps in services that the existing members are unable to do, or have little time to do. The primary care providers' previous knowledge and experience with OTs and PTs initially influenced this stage of the relationship-building. The team drew on this previous experience to extrapolate their initial understanding of the roles of occupational therapy and physical therapy in primary care. Most members of the team who participated in this study reported understanding of the physical therapy role from previous personal or professional experience in relation to musculoskeletal injury, while there was less consistency in having previous experience working with occupational therapy. Through the process of looking for help in the context of co-location, the team came to see how their current understanding of the role of occupational therapy and physical therapy in primary care was either similar or different from their previous perceptions of these roles. Regarding occupational therapy, this physician explains, “there's one area that I have come to see occupational therapy a little differently, and that was in relation to chronic pain.” (Physician 3).

The team's greatest **patient-focused** need identified in terms of **looking for help** was to better address chronic conditions such as persistent pain and mental health issues. Over time and with more experience, the members of the team began to become more aware of the role OTs and PTs could have in providing non-pharmacological treatment for individuals with chronic conditions such as chronic pain and depression. The primary care team was relieved to have a patient care need fulfilled when they understood that OTs were trained to address mental health issues, and that the PT and the OTs had skills in non-pharmacological and self-management approaches. The team welcomed OTs' and PT's use of cognitive behavioral therapy, behavioral activation, and motivational interviewing to enhance patient care, “the chronic pain patients and the disabled patients benefit tremendously from having multiple caregivers. And de-emphasizing the focus on their medication and increasing the focus on their own responsibility toward achieving greater health.” (Physician 1) Another physician expressed an expanded understanding of how the OTs' and PT's skills matched the patient needs for which they were **looking for help**, “…particularly around some of the behavioural support as well (referring to occupational therapy), you know. And just seeing that as more of a resource.” (Physician 2)

Early in the team development, this team saw a benefit of including OTs and PTs as members in the care plan when patient situations were complex or challenging. In this example, a physician who is **patient-focused** talks about **looking for help** to ensure a patient's best interests were being kept in mind despite needing to constrain a patient's autonomy.

“To collaborate on deciding when it was the right time to overstep his (the patient's) autonomy. …It was nice to have the team together to say like okay, are we all kind of ready to walk together to take this uncomfortable step.” (Physician 4)

3. **Fitting-In** meant the **patient-focused** team members developed their relationships using their co-location to interact and discover how the OTs and PT could broaden the team's services. These therapists considered the **fitting-in** process as part of their role when they began working at the primary care clinic. Therapists understood it was their responsibility to clarify which occupational therapy and physical therapy roles would be helpful to the team since these professions were new to primary care in Manitoba: “just collaborating with the interprofessional team and also defining our role in primary care.” (OT/PT 2)

The OTs and PT approached **fitting-in** by spending time understanding the team's needs in terms of patient care gaps. The team members had come to understand by **looking for help**, that the therapists could be helpful filling a gap with complex patients. Thus, the therapists often received referrals for patients that other providers described as being “stuck”. Patients that are “stuck” were those that seemed unable to engage or assume control of their health. After receiving referrals for these “stuck” patients, the OTs and PT used their skills to collaboratively set goals with patients.

“We get a lot of the stuck clients. What we're good at is getting them unstuck by setting very small realistic goals with clear action plans….. tangible goal setting is something that the rest of the team often doesn't engage in.” (OT/PT 3)

The OTs and PT determined they could contribute to patient care gaps for patients experiencing chronic pain. The therapists recognized that this gap was an area where the team was **looking for help** and used this specific health condition as an opportunity to show how they could **fit in** and enhance team care. This therapist described how the OT and PT focused their efforts on exploring needs and developing non-pharmacological interventions for patients with chronic pain:

“…initially we just looked at what the clientele was like and we thought we'd have some sort of role in chronic pain… we just looked at different handouts, we had an idea of developing modules, if I meet someone with chronic pain for the first time, I pull out module one and it's like making sure they've had general education – do they know what chronic pain is? Do they know what that means? Do they know that, that means they can continue activities? – like really basic education. Then module two will be looking at what coping strategies do they have and what are they interested in and then maybe a couple of modules that are actual coping strategies… maybe the physio does module one, someone else can pick up and do module (two).” (OT/PT 1)

The team, being **patient-focused**, expressed relief knowing that the OT and PT could provide the team with an evidence-based option of treating patients with chronic pain non-pharmacologically. This physician shared how they perceived that the PT was **fitting-in** by providing **patient-focused** strategies that were different than what a physician alone may be able to offer as treatment options, and that broadened the residents' understanding of how to provide high quality care for this patient population.

“I'm saying is that when the therapist is here, the resident learns the value of the physiotherapist in their knowledge of that but they also learn to utilize exercise in the management of the problem regardless of whether there is a physiotherapist here or not. Because otherwise we physicians would tend to focus on drugs and surgery.” (Physician 3)

This physician went on to share that the team is now recognizing that not offering non-pharmacological treatment options for issues like “chronic pain,” “musculoskeletal,” and “mental health complaints including depression” is “neglect”. (Physician 3)

A part of the process of **fitting-in** was for the OT and PT to negotiate areas of role overlap with the team. For example, **fitting in** was negotiated between OTs and the onsite mental health counselor, who already provided intervention approaches that were similar to the OTs. Prioritizing developing trusting and respecting relationships, the OTs while being **patient-focused** worked to **fit-in** with the counselor by discussing patient cases together and developing an understanding of how they could work together to best meet patient needs.

This example of negotiating roles to **fit-in** with the counselor demonstrates how informal communication became essential for learning about each other at a deeper level. Informal communication was particularly important to this team because it fundamentally valued relationship-building within the team, and with patients, as an essential component of providing high quality patient care. Informal communication allowed the team to be confident in the knowledge and abilities of the OTs and PT,

“…you can sit someone down and say this is what we do, this is my job description. That's good, but I think there is still an aspect of competence or I feel more confident in consulting someone once I've felt personally that they have their act together.” (Physician 1).

Once team members viewed the OTs and PT as competent, some of the team members began working to create a synergistic relationship. For example, the counselor and OTs began working closely together and one of the OTs anticipated that this relationship will develop further, “I've participated with the Shared Care counsellor in (delivering) a motivational interviewing (educational session) and we're probably going to do more of that. So that's kind of still (an) evolving piece of it.” (OT/PT 3)

**4. Growing Reciprocity** was demonstrated by this team as they grew to understand how the OTs and PT contributed to the team's shared goal of providing high quality health care. As the OTs and the PT got to know the team's needs (looking for help), and as the team's confidence in the therapists' competence to provide care grew (fitting-in), **reciprocity** amongst members began to grow, all the while being **patient-focused**. Again, the need for meaningful face-to-face two-way **patient-focused** communication continued to become more and more essential to developing trusting relationships. In this stage the team moved from the OTs and PT fulfilling the team's requests, to two-way communication where the OTs and PT were asked to address a patient's issue by using their own knowledge to determine an assessment and treatment plan. At this point, the team was wanting to work together inclusive of the therapists with all members deeply ascribing to the notion that this collaborative approach is how to deliver the best patient care. This physician describes such an interaction with the OT:

“…they will have either sent me a message and said, like I wonder if you might want to consider psychiatry referral to this patient because I'm concerned their functional barriers might improve with, with psychiatric treatment, with medical treatment. And then I might come back, you know, come back to the room and just have a conversation about like oh, do you, like did something come out in your assessment that I didn't notice? Or, you know, like can we work together? It's mostly about case planning and how we work together.” (Physician 4)

In the stage of **growing reciprocity**, team members themselves recognize the importance of considering the modes of communication that they use for interaction. While team members recognized that they could communicate in writing *via* the EMR, they viewed the telephone as a more satisfactory tool to have two-way interactions, and recognized that they shared even more information with face-to-face discussion because it allowed team members to integrate verbal and non-verbal communication. This sentiment about modes of communication was expressed by this physician:

“Because some of those could happen by phone. I know personally that the more information comes across when you have a conversation than when you write it down. There's even more that comes across when you have the conversation in person. That you're missing a lot of the non-verbal communication over the phone and sometimes you don't know what the silences mean exactly. And some of those would be exactly the kinds of things that you do want to communicate.” (Physician 3)

The team deemed face-to-face conversations especially helpful when they needed to have a difficult conversation. This physician described the **growing reciprocity** between the team and the OTs and PT with a shared goal of providing quality patient care during a face-to-face conversation:

“Because the occupational therapist doesn't want to come out and say, you're wasting my time with this referral. But you are constructively trying to send some of that same message, so tell me more about what you were hoping I was going to be able to do with this patient? And when the physician kind of flounders for a little bit, that is the message. And so that's a conversation that is, it's a difficult conversation. And having it by phone I'm not sure you would get the same, it's just, you can try to have that same conversation, it might be a confrontation. Whereas you might be able to have that same conversation in a constructive way if you had it in person.” (Physician 3)

The discovery of shared values is inherent to **growing reciprocity**. In this team, team members became aware of shared professional values, and also how the OTs and PT members could contribute to care related to these values. One example of this was that the team valued the therapists' unique perspectives related to function as a key indicator of overall health. A result of having an OT provide input to patient care, this physician recognized that their own expectations for patients may be incongruent with the patients' functional ability:

“…Because often times like we will feel like we're spinning our wheels with a patient who gets stuck in a situation. And sometimes the situation is a physical barrier. Almost always it's a combination of a physical barrier and a psychological barrier. And sometimes it's just a mental health barrier. But I think it's been really helpful to shift the way that I think about helping people towards what are some reasonable functional goals. Because, again, I think sometimes my functional expectations for people are, have them too high.” (Physician 4)

Another example of discovering shared values was a team member who discovered that the OTs and PT also felt that high-quality care should be patient-centered care with a focus on quality of life, “Even if it's like don't, accepting change, accepting differences in someone. But, again, just a bigger picture of what is going to make that person happy and their family comfortable and that kind of thing.” (Team Member 3)

For physicians specifically, their view of **growing reciprocity** included visioning how models of primary care teams with OTs and PTs should and should not be implemented. One example is that the physicians felt a responsibility to protect the health care system by limiting direct referrals to the OTs and PT. One physician explained that physicians should not have a “knee jerk reaction” where they begin to refer everyone to the OTs and PT because dealing with that issue “is in your skillset or should be in your skillset”. Some of the physicians underscored the importance of recognizing when some issues, “will get better on their own and we are not backlogging the system”. (Physician 4) In attempting to offer quality care while cutting costs, some physicians shared they would like to see capacity-building occur where physicians learn specific skills from the OTs and PT to be able to provide that service themselves rather than referring to a therapist. This physician provides an example of how they would like to see **growing reciprocity** to evolve in the clinic in a way that would increase physician capacity for musculoskeletal issues and reduce the number of PT referrals required.

“I would want to make sure that by having physio in our clinic what happens is that if I see somebody with an ankle sprain and I'm not sure what to do, I might refer to physio the first time and then the resident will go to that physio appointment, see what the physio does around recommendations for a recent ankle sprain and then the next ankle sprain that comes in that person doesn't get referred to physio because the resident knows what to do.” (Physician 4)

Another example of visioning that indicates **growing reciprocity** was considering new models of care that could be implemented that included OTs and PTs:

“Using a group visit model and utilizing OT and PT in terms of functional assessments and recommendations around whether they're using aids and things like that. Or behavioural support using them as a behavioural specialist within that context would be very helpful. So different groups you could have people of diverse issues coming together. You can have individuals that have similar issues like diabetes coming together for group management.” (Physician 2)

And even beyond this particular primary care clinic, members of the team considered the integration of occupational therapy and physical therapy within the health region more broadly:

“…I'm particularly interested in hopefully conducting a group, an interprofessional group care for the marginalized population downtown. …be also providing service to individuals who are either homeless or otherwise marginalized.” (Physician 2)

## Discussion

The purpose of this study was to explore interprofessional team relationship-building experiences using the ICRB. The findings both advance the development of the ICRB model by further explicating our understanding of the interprofessional team-building processes, as well as providing information that directs future clinical and research activities to advance interprofessional team-building. Our study findings reflect the perspectives of one interprofessional primary care team at a primary care clinic in a Canadian metropolitan center. The clinic integrated several other professionals in addition to the OTs and PT. At the time of the interviews, the OTs and PT had been part of the primary care team for 2 years. This primary care clinic was the first in this metropolitan center to integrate both occupational therapy and physical therapy services into a primary care team. The ICRB provided a relevant framework for examining this “young” team's relationship development.

This study helped to elucidate how the concept of **co-location** fits within the ICRB model. Co-location was the context in which the team built their interprofessional collaborative relationships rather than being a unique stage of relationship development. Co-location allowed for formal and informal interaction of team members and facilitated communication and understanding of each other's roles. Similar findings have been found in other settings and with other populations. Kennedy et al., found that co-location increased helping behaviors among surgical team members (26) while Rousseau et al. found that co-location was the primary predictor of youth mental health primary care team members' perceptions of successful interprofessional collaboration (27).

Considering how and when to support team co-location is particularly important in the context of the COVID-19 pandemic, which presents challenges for co-location. Gera reviewed the evidence on face-to-face and virtual teams and found that face-to-face teams were more satisfied, supportive and innovative (28). Virtual teams were more prone to conflicts, less satisfied and had inferior decision-making, though many teams were able to develop collaboration, trust and cohesion over time. As technology continues to evolve, it will be important to continue to examine primary care team relationship development in relation to virtual and physical co-location.

This study contributes to understanding the complexities of trust-building within a team and how it contributes to collaboration. In the early stages of the team-building process, specifically the stage of **looking for help**, team members wanted to know more about the therapist's values, specific skills and competence to determine how to work with them, and to gain enough trust for patient care sharing. Sangaleti et al. in a systematic review on teamwork in primary health care reported similar findings about the development of trust for care sharing, although they described challenging other professionals' competence as a team conflict (29). Conflicts in this context included situations where knowledge and skills were acquired from other professionals and a gap in understanding the role of other team members. In this study, we did not interpret this lack of role clarity as conflict as much as part of the process of **fitting-in** and **growing reciprocity** when working with other team members.

Further, in relation to trust-building, the stage of **growing reciprocity** is about team members interacting with one another, developing a deeper understanding of each other and of their shared professional values and how this interaction will contribute to quality patient care. Essential to this developmental stage is the knowledge that the team has a strong sense of cohesion that allows for different perspectives to not only be considered positively, but are thought to be an important aspect to delivering quality patient care (30). In this stage, the team could see how integrating occupational therapy and physical therapy could be a of value to the team's patient care. For example, the recognition of the OTs' and PT's understanding of the patient's overall function in a way that complemented and extended the physician's views was seen as an asset to the team.

This team provides insights into the concept of collaborative or shared leadership within interprofessional teams. This newly formed team was ground-breaking in its region in regards to the extensiveness of the primary care team, and had no local model regarding how to become a well-working interprofessional team. Although physicians were **patient-focused** and wanted to meet the patient needs, shared leadership with physicians was not well-established in this study. This finding is not surprising for this “young” team breaking ground in the area of primary care. However, exploring some of the hesitancies and barriers for collaborative or shared leadership is important as the Canadian Interprofessional Health Collaborative Framework identifies this type of leadership as an essential competency for interprofessional collaboration (31). “Shared leadership is an emergent and dynamic team phenomenon whereby leadership roles and influence are distributed among team members.” [(32), p. 5] Models of shared leadership have been found to benefit the whole team and are associated with increased staff satisfaction and engagement, while lowering staff turnover (33). Shared leadership may depend on the context and the situation, and can be formal or informal (34), with some contexts and situations requiring multiple leadership roles.

Hesitancy to share leadership was expressed in this study in the physicians' reluctance to relinquish control of patient care to other members of the team. Primary care physicians are known as the “gatekeepers” to other parts of the health care system (35), taking on the largest role in deciding on whether or not patients will be referred for additional services. In a recent survey of 61 PTs working in primary care in Ontario, PTs reported limits in their capacity because they needed to rely on patient referrals from other team members (11). Similarly, in a recent article family practice residents referred very few patients for occupational therapy and physical therapy services (20). While the OTs and PT did not explicitly comment on the reliance on team referrals in this study, the referral-based model of care may have impacted the potential for greater reciprocity in this setting. Recently the College of Family Physicians of Canada released the Patient's Medical Home suggesting that the primary care physician is the leader and delegates responsibilities to other team members perpetuating primary care hierarchies (36). These established hierarchies are contrary to collaborative or shared leadership (37) and neglect to acknowledge how team-based care can support the physician's ability to provide timely high-quality care. It is interesting to note that following the completion of this study, this team did move to a direct access model, rather than requiring physician referrals to occupational therapy and/or physical therapy, demonstrating that as modeled by the ICRB, the development of interprofessional teamwork is a process that develops over time.

## Limitations

Since this was a qualitative study, the reader needs to consider their own context in transferring the findings of this study. Being a secondary analysis, we applied the ICRB to the findings of a related study but did not ask team members directly about their understanding of the team's relationship-building process in relation to the ICRB. Similarly, it is unclear if data saturation was achieved as this was a secondary analysis of previously collected data where the data from the original study was used to answer the research question.

## Conclusion

The ICRB illustrates how collaborative relationship-building can be built in primary care teams to promote high quality patient care. Central to collaborative relationship-building is being **patient-focused** and having high quality face-to-face communication in addition to other communication methods. As primary care teams develop over time, they can enhance team satisfaction and patient care delivery by moving beyond interprofessional care to interprofessional leadership.

## Data Availability Statement

The data analyzed in this study is subject to the following licenses/restrictions: This data were interviews that were collected for the original study and were agreed to be kept confidential. Requests to access these datasets should be directed to cara.brown@umanitoba.ca.

## Ethics Statement

The studies involving human participants were reviewed and approved by University of Manitoba, Health Research Ethics Board (HS17336). The patients/participants provided their written informed consent to participate in this study.

## Author Contributions

PW led on conceptualization, study design, interpretation, drafting, reviewing and editing of manuscript, and contributed to data collection. CB led recruitment and data collection, contributed to the design, data analysis, interpretation, and reviewing and editing manuscript. MF contributed to the discussion and conclusion sections of the manuscript, reviewing, and editing of the final manuscript. LL contributed design, data collection, reviewing, and editing the manuscript. All authors contributed to the article and approved the submitted version.

## Funding

This work was supported by a ReHabilitation grant from the University of Manitoba, Rady Faculty of Health Sciences. The funding body had no role in the design or conduction of the study.

## Conflict of Interest

The authors declare that the research was conducted in the absence of any commercial or financial relationships that could be construed as a potential conflict of interest.

## Publisher's Note

All claims expressed in this article are solely those of the authors and do not necessarily represent those of their affiliated organizations, or those of the publisher, the editors and the reviewers. Any product that may be evaluated in this article, or claim that may be made by its manufacturer, is not guaranteed or endorsed by the publisher.
