# Supplementary material for: Interprofessional Collaborative Relationship-Building Model in Action in Primary Care: A Secondary Analysis
Source: Front Rehabil Sci. 2022 May 31;3:890001. doi: 10.3389/fresc.2022.890001 (PMC9397922; doi:10.3389/fresc.2022.890001)
Supplement: Supplementary file 1 [file Data_Sheet_1.PDF]

APPENDIX A Secondary Data Analysis Stages and Phases

Stage 1: Preparation Phase – Steps 1-7 (Assarroudi, 2018)

1. Acquisition of skills: Authors of this manuscript have formally completed graduate coursework on qualitative research approaches and conducted several qualitative studies.
2. Selection of appropriate sampling strategy: Individual team member interviews were selected from the available data which also included family medicine residents focus groups. Resident data was not included due to the limited time residents spend with the PC team.
3. Deciding on the focus of the analysis: Researchers elected to focus on relationship-building.
4. Developing the interview guide: Data used for this study included the participant responses to the interview questions used in the original study.
5. Conducting and transcribing the interviews: Eleven interviews that were audio-recorded and transcribed verbatim were used in this study.
6. Stating the unit of analysis: The unit of analysis was the team as the team's relationships with one another is the topic of interest.
7. Immersion in the data. All transcripts were read several times by the first and second author (CB & PW) exploring the utility for directed content analysis.

Stage 2: Organisation phase: Steps 8-15

8. Developing a formative categorization matrix: CB and PW used the ICRB for the analytic matrix including stages one to four and the two processes: patient-focused and communication strategies.

9. Theoretical definition of the main categories and subcategories: The theoretical definitions were taken from the description of the ICRB (Wener and Woodgate, 2016).
10. Determination of the coding rules for main categories: Each transcript was read and coded and then transferred to the appropriate stage or process section of the coding matrix.
11. Pre-testing of categorization matrix: Transcripts of the first three "team members" interviews were coded and analyzed into the ICRB coding matrix, carefully assessing the fit of the data. This pre-testing determined that the ICRB could be applied to this data set to provide insights into the team's relationship-building.
12. Choosing and specifying anchor samples: Samples quotes for each ICRB stage and process were selected from the first three transcripts
13. Performing the main data analysis: Meaning units related to the study questions were reviewed, summarized and given preliminary codes.
14. Inductive abstraction of main categories from preliminary codes: Preliminary codes were grouped according to their meaning and then grouped according to their similarities and differences to create generic categories.
15. Establishment of links between generic categories and main categories was achieved

<u>Stage 2:Reporting: Step 16</u>

16. Reporting all steps of directed qualitative content analysis and findings
